# Confined Students: A Visual-Emotional Analysis of Study and Rest Spaces in the Homes

**DOI:** 10.3390/ijerph18115506

**Published:** 2021-05-21

**Authors:** Teresa Cuerdo-Vilches, Miguel Ángel Navas-Martín

**Affiliations:** 1 Instituto de Ciencias de la Construcción Eduardo Torroja, Consejo Superior de Investigaciones Científicas (IETcc-CSIC), 28033 Madrid, Spain; 2 Escuela Nacional de Sanidad, Instituto de Salud Carlos III (ISCIII), 28029 Madrid, Spain; manavas@isciii.es

**Keywords:** COVID-19 lockdown, adolescents, secondary students, home perception, tele-study, Photovoice, sentiment analysis, qualitative, confinement, home spaces, children

## Abstract

Confinement was adopted globally as a containment measure to face the COVID pandemic declared by WHO on March 2020. In Spain, the State of Alarm was established for three months. This implied the interruption of educational activities, having a higher incidence for children, since teaching would not be resumed until the following academic year, in September. This, together with the confusing initial information about COVID-19 transmission between children and their families, has made them one of the groups most vulnerable. In this study, a qualitative approach is made to secondary school students (aged 12). They were asked to share their experiences about confinement from the perspective of the home spaces, in relation to two main tasks relevant in this period: the tele-study and their relaxing time and well-being. Using images and narratives with an abstract and emotional description, the response of 46 children was obtained. A sentiment analysis was carried out from their testimonies. Results suggest a greater availability of tele-study spaces with daylighting, mainly in bedrooms, with laptops. For leisure and rest spaces, sofas, beds, and cohabitant gathering were preferred. Written testimonials were mainly positive. Housing features and family cohesion condition their resilience in situations of uncertainty, like confinement.

## 1. Introduction

On 11 March 2020, the World Health Organization (WHO) declared the existence of a pandemic caused by a new coronavirus, called SARS-CoV-2 [1]. Countries adopted different public health measures to prevent the spread of COVID-19. The two main measures consisted of physical distancing and preventive isolation, as protection against the risk of contagion [2].

In Europe, Italy was the first country in the European Union to apply distancing measures. In Spain, the confinement was declared on 14 March 2020, when 5000 diagnosed cases were exceeded [3].

COVID-19 has affected the entire world population, including the very young. Some countries opted for containment measures or territorial shutdowns. One of the main measures carried out was the closure of schools and educational centers [2]. In Spain, at the request of the national government, face-to-face educational activities were suspended at all centers and levels. The Spanish government itself promoted the distance modality for teaching activities [4]. This situation lasted from mid-March until mid-June 2020, at which time the school year ended and the children were already on summer vacations.

During the first weeks of confinement, some studies detected a decrease in happiness and life satisfaction of people locked down, through cognitive discomfort and psychological distress [5]. Likewise, there was an increase in cases of stress, concentration difficulties, lack of enjoyment of daily activities, and lower ability for decision making and playing a significant role [6].

One of the groups that have suffered the psychological impact, with short-term implications, are children. Moreover, the problems are further aggravated in the case of children living in poverty. Situations such as instability at home, unstructured families, and especially a lack of parental control, promote the propensity to have more sedentary activities or spend more time in front of a screen. This was compounded by the closure of schools due to the lack of vital resources, normally provided by schools, during the state of confinement [7].

During the confinement for COVID-19, dwellings have apparently become a refuge for households, where people could carry out all the daily tasks but in totally unusual circumstances [8]. Depending on home characteristics and those of their spaces, they have brought with them very different sensations and emotions [9], from positive [10] to negative ones [11], even being able to develop or aggravate different mental illnesses, putting at greater risk the situation of vulnerability of many of these households [12].

The pandemic has reflected the digital inequalities that exist globally. Most low-income countries have the least access to the Internet [13]. These have also suffered the effects of education. In developing countries that have Internet connection problems, such as Pakistan, areas that have a high literacy, will be disadvantaged by not having an appropriate Internet connection that allows distance learning [14]. In more developed countries, Internet access and computer use are related to academic performance. With the pandemic, students with resources are more protected against vulnerability than students with fewer resources, who may see their level of education compromised [15].

In Spain, most students have mobile devices and an Internet connection. Although, there is evidence that the fact of having technology at home is not enough reason for students to follow classes with guarantees for learning. Some students state that they do not have sufficient resources [16].

During all this time, many studies have been carried out to determine the effects of confinement, both studies through online questionnaires, longitudinal or population studies, especially related to mental health. A study conducted in Singapore related the neurophysiological response to urban spaces [17]. In the UK, a number of longitudinal health-related household studies were conducted before and during the initial phase of the blockade or in different waves [18,19]. In Italy, a longitudinal and cross-sectional study was conducted on the impact of the COVID-19 closure on mental health [20].

Other studies have focused more on young people with the pandemic (COVID-19 pandemic). In Switzerland, a longitudinal study was carried out on the dimensions of social networks and the mental health of students before and during confinement [21]. In Italy, a longitudinal observational lifestyle study with children and adolescents with obesity was undertaken [22]. In the Netherlands, a longitudinal study was conducted with university students to investigate the impact of the block on mental health [23].

To date, there are no studies where students express their experiences regarding the tele-study spaces, used during the confinement period by COVID-19, and in general, the perception of both this space and other spaces in the house in this context. The aim of this study is to better understand how adolescents perceive the home spaces regarding their main tasks during the lockdown. More specifically, the objectives are: (1) analyze how children describe these spaces visually and in written form and (2) establish traits from the emotional state, linked to these descriptions and thus, to the own space perceptions, and if possible, to children’s own situation of confinement.

The research questions on which the request for this information was based were the following: Q1: How do adolescent students perceive their tele-study spaces? Q2: What are the most comfortable spaces for these adolescent students, given the situation of confinement at home, and why? and Q3: Is it possible to detect traits of emotional state from their narratives and testimonies related to those space descriptions?

## 2. Materials and Methods

There are many methods that allow participants to be guided to express themselves through images, for example using Photovoice [24]. Photovoice is a methodology that can be used in different communities [25].

Photovoice is based on the community-based participatory research (CBPR) approach [26], where, in addition, individual reflection is promoted and does not need the guidance of the main researcher [27], supporting the discourse based on images created by participants, with contextual narrative [28].

There exist different methodological adaptations that are applied to Photovoice for its implementation, as a result of its versatility. For example, it has been adapted to be applied over the Internet [29]. Thus, for example, a study with people with chronic diseases asked its participants to upload their photos to the Instagram social network [30]. Other adaptations are based on the use of other graphic expressions to provoke individual and/or group discourse, such as photo-elicitation [31], which promotes the use of photographs taken from other sources, or the use of other graphics, such as drawings by the participants themselves [32], validating its use as detection even of other problems [33]. This would allow participation without the need for cameras [27]. This adaptation has been used to know, for example, the criterion of comfort in teaching classrooms by adolescent students, in southern Europe [34], based on the capacity of the non-adult population, of the age range 12–16 years, to generally develop, in an acceptable way, abstract descriptions about the built environment, its characteristics, and the sensations and emotions that their relationship with these spaces provoke [35].

The Photovoice method has been widely used in general to address problems of a community nature, especially those less favored, since it promotes the awareness of the participants towards their reality, to empower them towards the search for improvements and decision-making communication [36]. This explains its application to problems related to Social Determinants in Health, and more specifically, to problems associated with learning [25] and even to mental health [37,38].

This study is based on the power of the image to show certain home spaces (workspaces and most comfortable ones) and provoke narrative discourses in the authors that expose the purpose and intention of the respective snapshots. This study was carried out using an online form, for obvious reasons related to confinement, where two photographs were requested, and relative keywords and the answers to certain questions, based on the SHOWeD forms [39].

### 2.1. Participants and Procedures

To recruit the participants, a school was contacted in Madrid, Spain. This school, which has approximately 2000 students between the ages of 3 and 18, covers all stages of teaching, compulsory or not, from early childhood education to university. Due to the age range, it was decided to launch it in the first year of secondary, equivalent to 11–12-year-olds. By contacting through the Directorate of the Center, to obtain their authorization and act as facilitator, it was promoted as an activity from the Spanish Language subject (without being evaluated), where previously in the contents of the subject the abstract description of spaces, both objective and emotional, was explained. With the help of the subject teachers, a preliminary group session was organized to explain the task online, in which they themselves briefly explain the action. As part of the explanatory pre-session, students were encouraged not to take photographs that could reveal the identity of any person, themselves or others, directly or through recognizable objects. Seriousness is requested when participating and truthfulness and commitment in their responses. Sufficient and pertinent information was previously sent to the parents, so that they could decline their children’s participation if they did not agree.

The data was collected from 1 June to 18 June, within the period of confinement decreed by the Spanish government [4], which lasted from 14 March to 21 June 2020 [40].

As the participating students were the same age, no sociodemographic data was requested, so as not to hurt potential sensitivities. The treatment of the information obtained had always been anonymous, since once the teachers provided the web link of the online form, no one controlled the access of the students, and a password or something that revealed their identity was not requested; personal data, including the IP, was not recorded.

### 2.2. Data Collection

The online form that was offered to secondary school students arose as an adaptation of the (COVID-HAB) project, on Confinement by COVID-19, housing and habitability [8], developed in Spain during the confinement period in the spring of 2020. This project was funded by the Spanish National Research Council (CSIC) and obtained the approval of its Ethics Committee. Its general qualitative form, adapted from the Photovoice technique [41], was simplified to adapt it to secondary-school students, thus limiting the number of photographs; the language and the number of questions associated with each photograph requested were also adjusted. Questions on sociodemographic data were also eliminated, because this information was not relevant enough to potentially lose part of the participation due to violating privacy in compromised aspects, such as place of origin or place of residence; these could lead to student identification.

After a virtual study presentation by the Spanish-Language teachers, as a voluntary and anonymous task, the students were provided with a web link that they could access from any device with an internet connection (computer, laptop, mobile, or tablet), taking into account that they had to upload two photos. If the device did not have a camera, they would previously have to transfer between devices to fill in the online form.

The SurveyMonkey^®^ online platform was used to facilitate the data collection questionnaire for the participants. This was characterized by having a friendly design, being easily adaptable to any device with an Internet connection. The form was designed as a questionnaire with thirteen entries, the first being the consent to participate in the study. The remaining ones are included in 2 blocks, corresponding to: (1) a tele-study space at home and (2) the most comfortable space (which you like the most). Each block had corresponding entries related to a photograph, a group of 3 tags or keywords, and 4 related questions.

The tags were asked to classify and thus, better categorize the related photos, three for the tele-study space and the other three for the most comfortable one. The questions for each photo were:

- What does this photo show?
- Why did you take this photo?
- What does this photo express about your life now, during the lockdown?
- What message could this photo give other boys and girls aged like you, to improve their lives?

These questions allowed children to contextualize the photos, their description, and the intention to take them. The answers given were used to analyze emotional aspects (especially for the two latter).

### 2.3. Data Analysis

#### 2.3.1. Graphic Analysis: Categorization of Photographs

For the analysis of the images, the qualitative analysis software NVivo Release 1.3 was used. All photos were coded for their subsequent categorization, using an alphanumeric code.

#### 2.3.2. Textual Analysis I: Word Frequencies and Clouds

Word clouds were created for textual analysis. These clouds facilitated the analysis of the text and made it possible to identify the most relevant topics of the analyzed texts. Visually, the words with the highest frequency were shown larger [42]. For the realization of word clouds, their frequencies were calculated, for which empty words were eliminated (those with a minimum length of 3 letters, and applying similarity for derived words). These clouds were made for both of the answers to open questions about the photos as well as to the tags or keywords. For the qualitative analysis, the NVivo Release 1.3 software (QSR International–Americas-, Burlington, CT, USA) was used.

#### 2.3.3. Textual Analysis II: Verbatims

The most significant verbatims were selected. The sentences of simple constructions were discarded, and three verbatims were selected as a sample of each of the questions asked. NVivo release 1.3 software was used for selection.

#### 2.3.4. Textual Analysis III: Sentiment Analysis or Opinion Mining (Polarity)

Sentiment analysis is the field of study that allows us to analyze feelings, opinions, appreciations, evaluations, emotions, and attitudes that people transmit. Among the different forms of transmission of opinions is the use of written language. In the last twenty years, there has been a growing interest in these analyses, both in the areas of research and business [43].

The information available in a text can generally be classified into two categories: facts and opinions. Facts are characterized by being objective expressions, while opinions are subjective. Opinions usually describe feelings or evaluations that people make of their expressions. These expressions usually convey positive or negative feelings [44].

The use of sentiment analysis can be applied in most areas of human activities. Opinions affect almost all facets of our lives and influence our behaviors behaviors [43]. Among the different applications, the education sector stands out. In the school environment it is used to know the feeling from the student comments [45], to improve the quality of the education system and the monitoring of the teaching level from teachers seen by the students themselves, and on student learning [46,47].

Through sentiment analysis, emotions or opinions can be detected from a set of data. It allows one to measure the polarity of the attitudes expressed in a text. Its use facilitates better decision-making on public opinions, in addition to providing valuable information, especially in the analysis of social networks [48,49]. There are three kinds of polarity: negative, neutral, and positive. Normally, the analysis systems use a scoring scale, ranging from -1 to 1, where a negative score corresponds to a negative sentiment; zero is considered a neutral sentiment and a positive score corresponds to a positive sentiment [48,49,50].

Tools based on automated analysis algorithms are used to perform sentiment analysis. There are a wide variety of sentiment analyzers, among them are Textblob, SentiWordNet, and SentimentR [48,50,51,52]. Textblob is developed in Python and is open access; it is based on the use of basic features of elements of Natural Language Processing (NLP). It is recommended to analyze short texts, such as tweets [48,50]. SentimentR is a package implemented in R and is based on the use of the Jockers–Rinker lexicon that is characterized by assigning the polarity of the words based on the strings with changing valences [52].

In this study, and in order to obtain greater precision in the sentiment analysis [48], more than one analyzer was used. Specifically, the Textblob sentiment analyzers were used for R [53] and SentimentR [54]. For this, the open-source R environment was used for statistical computing [49] using the package libraries of both parsers. These analyzers were chosen because they are free tools and due to their availability for use in R.

To obtain the polarity of the texts, the responses of each student were analyzed. Through the tools available in the installed libraries, the translation from Spanish to English was performed first, and then the polarity was calculated through both analyzers. For the quantification of polarity, an average of both results was carried out, taking into account that both programs use the same scale (−1 to 1). This allowed us to quantify the opinions in three groups: negative (<0), neutral (0), and positive (>0).

## 3. Results

Initially 66 responses were collected, 46 being valid and fully answered. Of these 46 completed forms, 137 labels and 46 photos were collected for the first block (the assessment of tele-study space), and 132 labels and 42 photos for the second block (the evaluation of the most comfortable space), with their corresponding answers to the related questions.

### 3.1. Selection of Photographs

**Figure 1:**
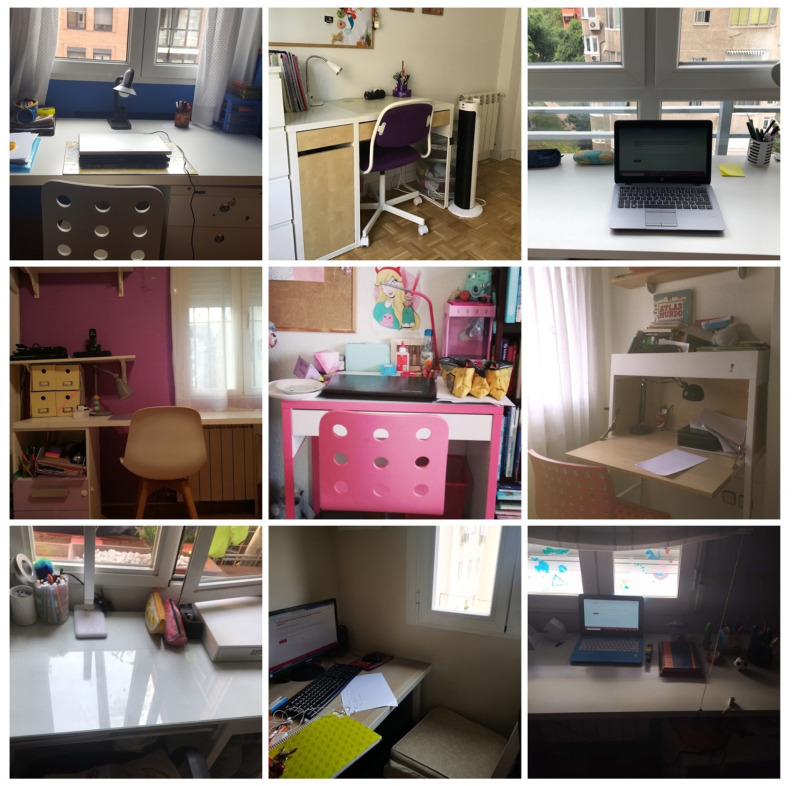
Exclusive or usual spaces for tele-studying, many equipped with computers.

**Figure 2:**
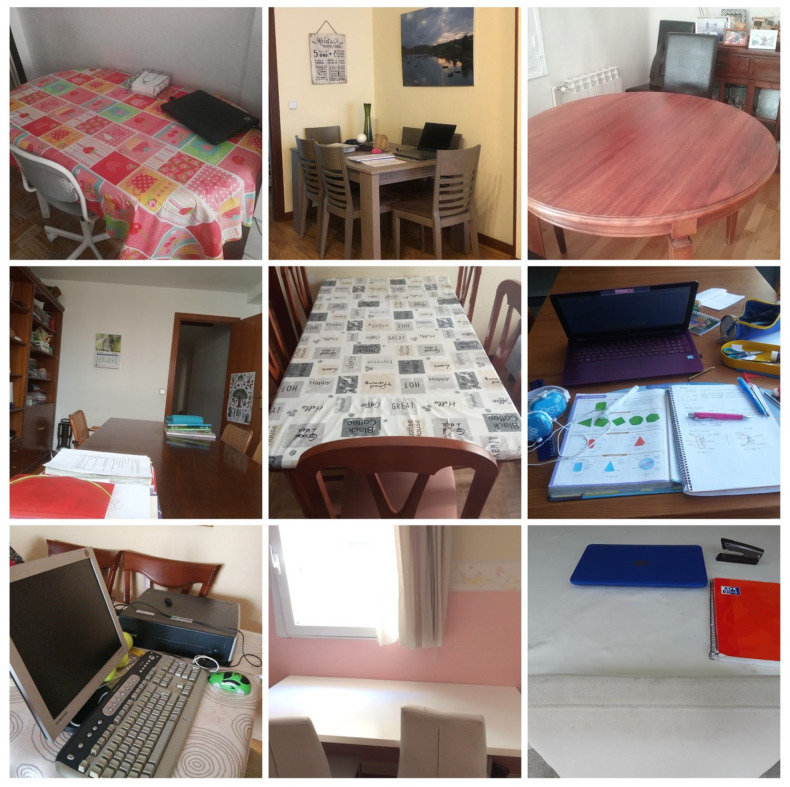
Eventual or roaming spaces for tele-studying, in different home areas.

A total of 88 photos were obtained between the two blocks. Among all these photographs, the nine most significant were selected by theme. According to the categorization carried out on the photographs of the first block, relative to the tele-study space, distance work stood out both in exclusive spaces—studios—or in shared use on a regular basis—kids’ bedrooms—(Figure 1), as well as their use in shared spaces sporadically or occasionally, such as living rooms and dining rooms (Figure 2). Many of them required the use of computers, although some of the photos did not show them.

**Figure 3:**
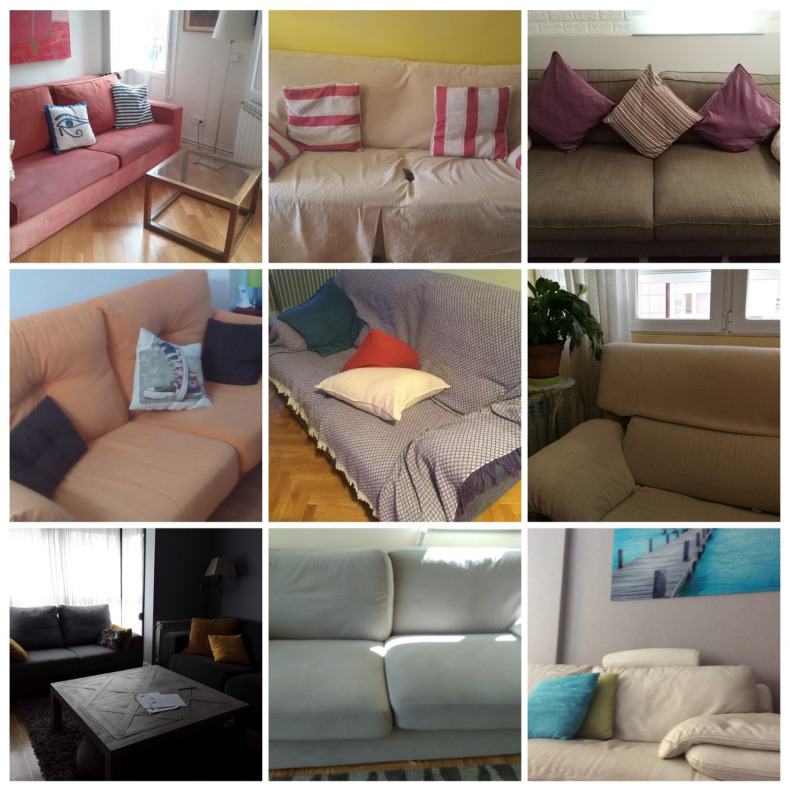
Comfortable spaces, referring to living rooms, and specifically, sofas.

**Figure 4:**
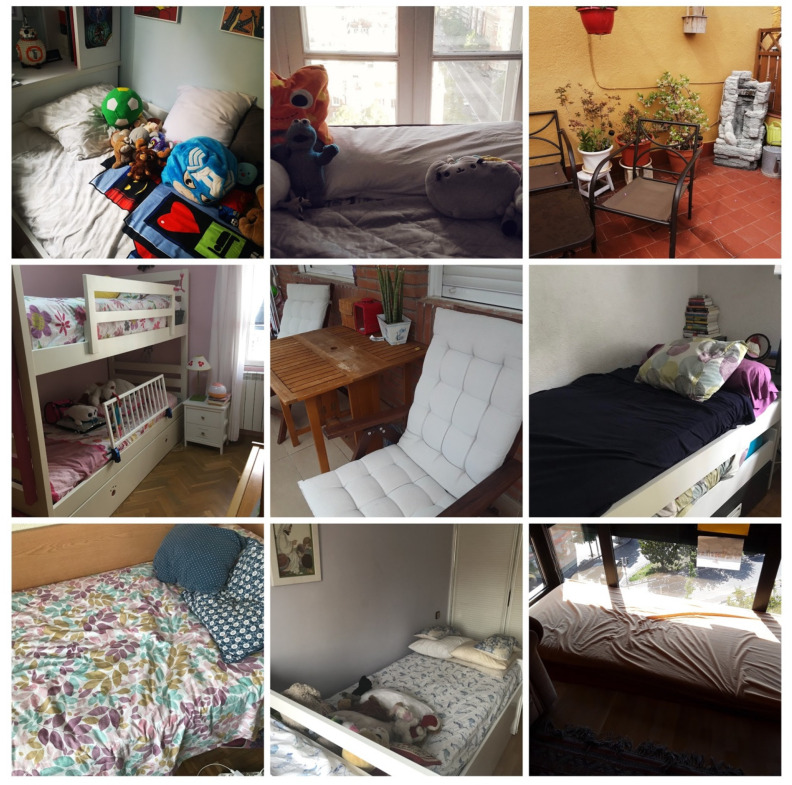
Comfortable spaces, referring to bedrooms, beds, and outdoor spaces.

Regarding the second block, on the most comfortable spaces in the house, the photographs of sofas (Figure 3) and beds (Figure 4) stood out.

### 3.2. Textual Analysis

#### 3.2.1. Categorization of Photos

(1) Block related to tele-study spaces. Images analyzed: 46.

Table 1 presents the most frequent characteristics observed in the photos and narratives related to telework spaces and resources.

Most have exclusive or usual space, natural light, and a computer, the most common being the laptop. Almost all of them have ordered spaces.

(2) Block related to the most comfortable spaces in the home. Number of analyzed photos: 42.

Table 2 presents the most frequent characteristics observed both in the photos and the testimonies related to the most comfortable spaces.

Two categories stood out, one was the use of a sofa in shared places and beds in rooms.

#### 3.2.2. Word Frequencies and Clouds

In Table 3, word clouds referred to photo tags are shown, for each content block. Table 4 includes the most frequent words related to narratives on telework, whilst Table 5 shows the most used ones on the most comfortable spaces in the homes.

#### 3.2.3. Most Relevant Verbatims

For each of the questions, a maximum of 46 responses were obtained. The three most significant verbatims were selected from the responses to each of the questions (Table 6).

### 3.3. Sentiment Analysis

Regarding the sentiment analysis, the descriptive questions (Q4, Q5, Q10, and Q11) were discarded. The analysis of the questions was carried out in which the participant was explicitly asked for an opinion on the topic (block) proposed for each question (Q6 and Q7, for block 1; Q12 and Q13, for block 2).

Of the four questions as a whole (Q6, Q7, Q12 and Q13), it was observed that most of the opinions were positive, 115 (64.25%), neutral to a lesser extent, 38 (21.22%), and negative, 26 (14.53%).

Table 7 shows the polarity from responses to these questions, frequencies and percentages. 

**Figure 5:**
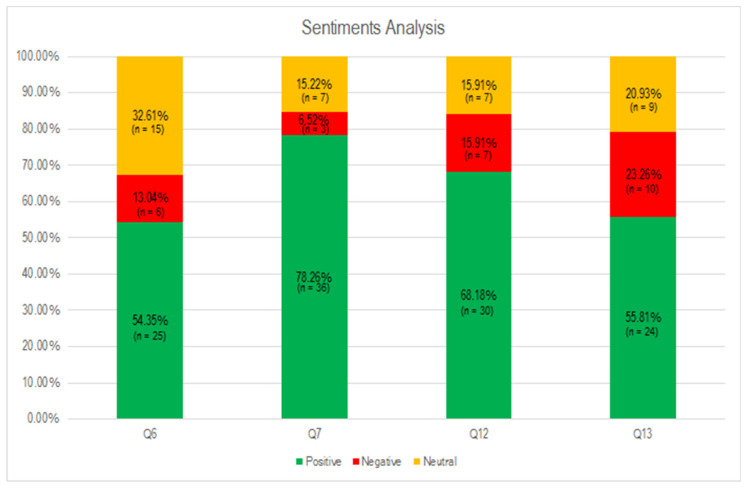
Sentiment analysis.

Regarding the individual results per question, in each of them the answers showed a tendency towards positive polarity. The results of question Q7 stand out with 38 positive responses (78.26%) and question Q12, with 30 positive responses (68.18%). Figure 5 includes the distribution of positive, negative and neutral responses from the sample.

## 4. Discussion

This study was designed to explore the housing spatial and emotional perception by secondary-school students, forced to tele-study at home due to the circumstances derived from confinement by COVID-19. To do this, researchers contacted a school located in Madrid (Spain), where the voluntary participation of four class groups was achieved, conducted through the Spanish language subject, corresponding to the first year of compulsory secondary education. Of the one hundred potential participants, 66 answers were obtained, a total of 46 being valid. These students were between 11 and 12 years old. In this unusual situation, the household as a reference and the dwelling as a bulwark played a decisive role in the emotional stability of the students. There is evidence that indicates the importance of the participation of the family unit for the development of learning activities within the home. In the case of Spain, various platforms were provided to promote co-creation by all agents (teachers, students, and parents), during the learning process [55]. According to the comprehensive content analysis, the family was an important element in the responses of the students. Although many students had exclusive or habitual spaces, others used spaces such as living or dining rooms, which were shared with other members of the family. The importance given to the family nucleus by these adolescents, although not clearly evident in the photographs, was outlined in tags and the narrative that accompanied them.

On the other hand, the direct relationship between health and well-being with academic performance is observed [56]. The home has been, in general terms, the refuge of the students during the confinement. Bearing in mind that housing is a social determinant in health [57], a poor habitability in the home can put the health of the occupants at risk [58], and specifically, mental health [59]. Therefore, the home features are important factors to take into account. Among those features to define healthy housing, some are highlighted: construction materials and furniture that are environment and health respectful; overall comfort (hygrothermal, acoustic, and visual); lighting (specially daylighting); indoor air quality; safety; and social quality [60].

After observing the photos uploaded by participants on the tele-study space, almost two thirds of them were located in what seemed to be youth bedrooms, giving the idea of having habitual spaces for study, exclusively. The order in almost all of them also stands out. This aspect, although many refer to it in their narratives, may be due to some type of adult intervention during the activity.

Another aspect to highlight is the high rate of natural light present in the study places, taking into account that they are mostly bedrooms. This was very positive, as daylighting has clear beneficial effects on biorhythms, personal development, and other factors that directly affect health [61]. A healthy habitability is also related to indoor environmental quality, of which good lighting is a part.

Regarding digital resources, in general the participants used computers, mainly laptops. Those who did not show computers to tele-study in their photographs may be because they shared computers with their relatives or they followed the classes from other types of mobile devices. Therefore, having a good Internet connection and coverage throughout the home is important to facilitate work as well as leisure.

According to the terms most used to refer to tele-study spaces, the reference to qualities such as comfortable, bright, or spacious stands out. Since they clearly show spatial preferences, psychology literature relates this kind of attribute to positive feelings. By contrast, narrow, uncomfortable, messy, or flawed spaces are often associated with negative feelings [11]. In fact, among the emotional consequences found in studies related to confinement, anxiety and depression were highlighted among the youngest [55].

In all the result sources analyzed in the study (photos, tags, and testimonies), the need for rest stands out, linked to what children understood as “most comfortable spaces”, with the bed or the sofa being the preferred elements. Therefore, the bedroom and living room locations were almost equal. This shows that, despite being at home all the time, many of them needed moments of disconnection in their daily lives. To a lesser extent, other locations were named, such as open spaces to the outside (terraces, patios), clearly referring to the need for contact with the outside and/or with nature, as a source of well-being and emotional stability [62,63].

With respect to the preferences detected, the sofa stood out also because it represented the meeting place with other family members, reported on multiple occasions by young people to be linked to tranquility, disconnection, meetings, and leisure. Indeed, it had been the most social moment, that they experienced in these circumstances, standing out against other expected words, such as those terms on technological leisure, for example [64].

As for the polarity of the opinions made by the students, the majority were positive messages. To a lesser extent, responses with a negative polarity were found. This leads to suppose that, despite the state of confinement, having spent it at home with their relatives, they could condition the positive feeling by a good parenting practice that allowed reinforcing family ties and the psychological needs of the children [65].

As main limitations of the study, we can highlight, on the one hand, those related to the type of qualitative research, which also constitutes its own wealth. Qualitative research cannot be replicable, nor is it probabilistic, so its results do not imply inference about the entire population [66]. However, this type of research promotes the study of a certain phenomenon in a detailed way, which allows knowing quality information about the reality experienced by a certain community [67]. Furthermore, with the analysis of this information, other research techniques can be approached later. Using it as a basis, future research could delve into the phenomenon and with greater scope and level of adjustment to certain ways of living this experience.

Regarding the limitations inherent to the analysis of domestic spaces, it would have been interesting to obtain more detailed information on indoor environmental and habitability aspects [68]. They are related to the quality and nature of lighting; natural ventilation; the time dedication to main tasks; the spatial and activity distribution of the household members through separate hours per day/night, and week/weekend days. Another interesting aspect would have been ergonomics and furniture [69]. Yet another would have been the actual availability and alternate use scheduling of different electronic devices by different cohabitants, as well as the time dedication, in order to evaluate the suitability of these children’s resources. Similarly, other aspects of general comfort, such as environmental quality (in terms of indoor air quality, perception of bad odors or stuffy environment, thermal comfort, use of heating systems, or noise insulation), would have been very interesting to evaluate, around the quality of these domestic spaces, and especially those related to the work environment of adolescents.

Valuations on topics such as the available bandwidth, the stability of the Internet connection, and the access to digital resources (also taking into account the alternation of use with other household members or their quality), are relevant to establish whether there really is a digital divide, which would be a handicap for education and the proper development of adolescents, in long periods of learning such as this confinement.

It could also be relevant to consider, as a future research line, contacting schools in vulnerable and non-vulnerable areas, to access and evaluate different households’ situations that might surround children and assess whether such aspect would alter their perception of family protection, as well as the need to feel their relatives closer in a context of confinement. To do that, a multidisciplinary team, a more specific research design, and contacts acting as facilitators could be necessary to generate a climate of trust to be able to carry it out with guarantees. In this research, specific aspects to stability of family nucleus were not assessed to avoid any sensitive information; thus, it is out of this study scope.

On the other hand, this study contributes to the legitimacy of adolescents to communicate their experiences, needs, and preferences of building spaces [34]. A lack of studies focused on the visual and qualitative description of domestic environments, in confinement, and even more focused on the youngest children, has been detected.

The relevance of the characteristics of the home [70] and the family environment are decisive in the lives of adolescents [65] and even more so when addressing the dedication to study, do leisure activities, and rest in the home in a confined context.

The positive perception of workspaces is linked to the possibility of spatial isolation for greater concentration, with suitable resources, in a quality environment, with natural light, and, where possible, comfortable furniture.

Similarly, during rest and leisure time, comfortable furniture is used, highlighting the relation to bedrooms and living rooms, claiming on this occasion the presence of other cohabitants for the moment of social interaction.

These keys are decisive when designing domestic spaces, since aspects such as the density of occupation per useful room surface, the environmental quality of the spaces, and the possibility of isolating/gathering when required, are fundamental to guarantee a correct performance of these activities, as well as an emotional balance, relevant in periods of uncertainty such as confinement. In this sense, housing contemporary designs establish multiple dialogues that allow flexible spaces to cover the life cycle of the households, as well as other adaptive requirements, like when confined [71,72,73].

## 5. Conclusions

The relevance of the home, its characteristics, as well as the guarantee of supplies and necessary digital resources have been key in this period of confinement for the population and their health, both physical and mental. In particular, they have been so for young people, who have had to carry out the tele-study. It also highlights the importance of ubiquitous learning, in addition to digital skills, both for young people and for other agents involved (teachers, parents). The qualities of both the home and the dwelling have been crucial to the emotional stability of adolescents. Having pleasant spatial and emotional environments favors mental well-being and a sense of security in youth. In this study, the need to distinguish work from leisure, disconnection, or rest has been perceived. In the circumstances in which both tasks occurred in the same physical space, the differentiating elements (desk-bed, for example) were indicated, to show the change of task. Once again, the moment of rest, leisure, and disconnection is indicated, which is not only linked to relaxation, as opposed to daily work (tele-study), but also to the moment of social gathering and family exchange, valuing more positively the spaces where they had such encounters. The adaptive or resilient capacity of young people is noteworthy, who have been able to see in general terms the positive of the exceptional situation experienced, as shown by the polarity analysis and some verbatims.

Long-term studies will have to be established to assess whether this trend is maintained, and, if a similar situation were repeated, or if it had been prolonged over time, what would have been the emotional evaluations of these young people.

Therefore, the future lines of research that are proposed are several: on the one hand, from the scientific and academic field, a representative study must be established, with probabilistic and random sampling, such that it evaluates telework spaces in general, in the homes, and more specifically, in the case of children, their tele-study space, from all the aspects previously exposed. Although in schools and tertiary buildings (workplaces) there are protocols, standards, and guidelines to achieve healthy, comfortable, and pleasant environments for workers, in housing this type of measure has not been applied, especially when resorting to them unexpectedly and abruptly. From now on, when the foundations of the lived experience are being laid, it is recommended to deepen this knowledge, as well as the establishment of contingency plans, which guarantee the supplies of both energy resources, internet, and the digitization of homes, to overcome the digital divide, the comfort, and quality of dwellings, assuming telework and tele-study as new domestic tasks.

In this sense, the duty of architects and planners is to include in housing discourses designs that solve the new needs that have arisen after reflection on what the pandemic confinement in housing has meant [74,75]. Among them is the new demand for quality spaces for teleworking and tele-study, where all the factors already mentioned related to indoor environmental quality, health [66,67,68,69], and respect for the environment, as well as access to supplies as a universal right, are present, without undermining the right to affordable housing.

Finally, guidelines aimed at making the most of daylighting, a correct postural hygiene, and the spatial and organizational optimization of domestic spaces and resources, could serve families in case situations like this arise in the future. More specifically, it would be useful if they could acquire a great adaptive capacity to face the situation (resilience) and have clear notions that they can apply this capacity to the best of their ability. These recommendations could also be adapted for a younger target audience.

## Figures and Tables

**Table 1:** Categorization of pictures’ content related to tele-study spaces.

Concept/Category	Subcategory	Frequency
Lighting	Artificial	6
	Natural	34
Computer	Laptop	19
	Personal computer	7
	No computer	16
Spatial organization	Messy	3
	Tidy-up	38
Location-room	Bedroom	24
	Living room	13

**Table 2:** Categorization of pictures’ content related to the most comfortable space.

Concept/Category	Subcategory	Frequency
Lighting	Artificial	4
	Natural	38
Preferences	Sofa	19
	Bed	12
	Outdoors	5
	Computer	2
	Television	2
	Reading	1
	Cooking	1
Location	Bedroom	17
	Living room	16
	Terrace	5
	Kitchen	1

**Table 3:** Word clouds referred to tags 1 (Tele-study) and tags 2 (Most comfortable space).

Word Clouds	Words	Frequency	Percentage
**Tags 1. Tele-Study**			
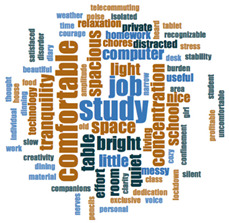	study	10	6.58
	comfortable	9	5.92
	job	8	5.26
	concentration	5	3.29
	space	5	3.29
	spacious	5	3.29
	table	5	3.29
	tranquility	5	3.29
	bright	5	3.29
**Tags 2. Most comfortable space**			
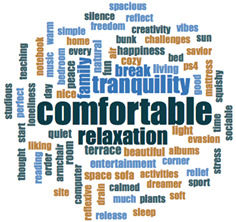	comfortable	22	15.60
	tranquility	12	8.51
	relaxation	11	7.80
	family	6	4.26
	break	5	3.55
	terrace	3	2.13

**Table 4:** Word clouds referred to picture-related questions on tele-study spaces at home.

Block 1: Tele-Study. Word Clouds for Related Questions.			
	Word	Frequency	Percentage
Q4: What does this photo show?			
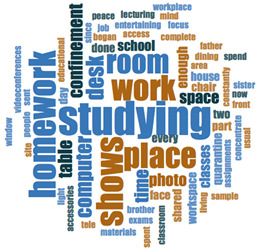	studying	16	9.04
	homework	12	6.78
	shows	12	6.78
	Work	11	6.21
	Place	11	6.21
	Room	10	5.65
	Desk	7	3.95
	computer	6	3.39
	confinement	5	2.82
	table	5	2.82
	time	5	2.82
Q5: Why did you take this photo?			
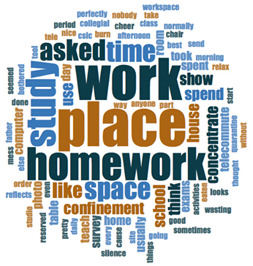	place	14	8.24
	work	13	7.65
	homework	10	5.88
	study	9	5.29
	space	6	3.53
	time	6	3.53
	asked	6	3.53
	like	4	2.35
Q6: What does this photo express about your life now, during the lockdown?			
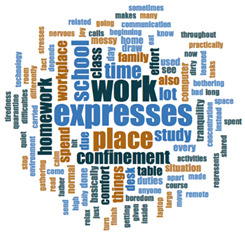	expresses	13	6.10
	work	13	6.10
	place	10	4.69
	school	8	3.76
	time	7	3.29
	confinement	6	2.82
	homework	6	2.82
	lot	5	2.35
	study	5	2.35
Q7: What message could this photo give other boys and girls aged like you, to improve their lives?			
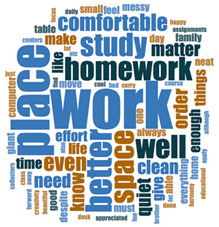	work	15	5.49
	Place	12	4.40
	better	8	2.93
	homework	7	2.56
	space	7	2.56
	study	7	2.56
	well	7	2.56
	comfortable	5	1.83
	even	5	1.83

**Table 5:** Word clouds referred to picture-related questions on most comfortable spaces at home.

Block 2: Most Comfortable Space. Word Clouds for Related Questions.			
Word Cloud	Word	Frequency	Percentage
Q10: What does this photo show?			
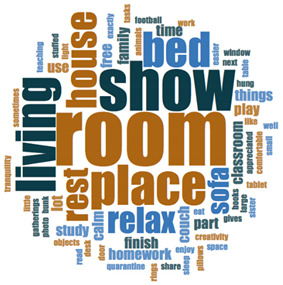	room	13	9.09
	show	10	6.99
	living	8	5.59
	place	8	5.59
	bed	7	4.90
	house	6	4.20
	terrace	6	4.20
	relax	5	3.50
	rest	5	3.50
	sofa	4	2.80
Q11: Why did you take this photo?			
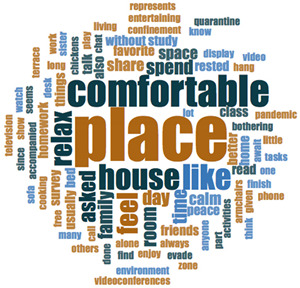	place	22	11.64
	comfortable	12	6.35
	house	9	4.76
	like	9	4.76
	relax	7	3.70
	feel	7	3.70
	asked	5	2.65
	day	4	2.12
	family	4	2.12
	room	4	2.12
	spend	4	2.12
	time	4	2.12
Q12: What does this photo express about your life now, during the lockdown?			
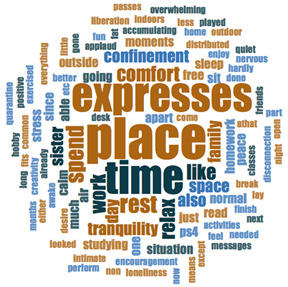	place	14	6.28
	expresses	11	4.93
	time	11	4.93
	rest	5	2.24
	spend	5	2.24
	like	4	1.79
	work	4	1.79
	comfort	4	1.79
	relax	4	1.79
Q13: What message could this photo give other boys and girls aged like you, to improve their lives?			
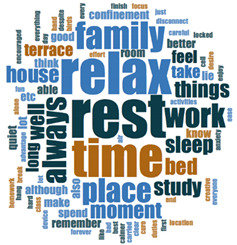	rest	12	4.60
	relax	11	4.21
	time	10	3.83
	family	7	2.68
	always	6	2.30
	place	6	2.30
	work	6	2.30
	important	5	1.92
	sometimes	5	1.92

**Table 6:** Selection of verbatims from answers to each question about the photographs.

	Block 1: Tele-Study Questions
**Q4**	*“It shows my work space that I have had during the quarantine where I have done all the school work that I have been sent to do during the confinement”.* *“The dining room table, where most of the time I have done homework and work”* *“My room and now, my place of work and where I do my homework and chores”*
**Q5**	*“Because it’s a place where I can study without being bothered by anyone”.* *“Because it is the space or room where I work and do my homework and exams”* *“Because that’s where I’ve spent most of my confinement”*
**Q6**	*“That I am working daily on a computer instead of going to classes due to the situation”.* *“Here I have carried out my tasks and I have been long time during confinement”.* *“That you work a lot more now than at school”*
**Q7**	*“That despite having many distractions when doing homework (like a little brother), you have to go forward, make an effort to be able to take the course”.* *“Well, you need a clean and organized space to study, concentrate and carry out your day-to-day tasks”.* *“That when you are doing homework or exams, it is better to be in a comfortable place that you like to focus better”.*
	**Block 2: Most-comfortable space questions**
**Q10**	*“The living room of my house, where, when I have finished my homework, I go to relax”* *“It shows my living room, where I relax with my family”.* *“The place where I rest and we do family meetings”.*
**Q11**	*“Because it is the place that has given me the most peace during the confinement because I have done many things there during the pandemic”* *“Because it is the place in the house where I feel most comfortable and where I spend my free time and enjoy my family”.* *“Because my bed is the only place in the house where I can be quiet and without anyone bothering me”*
**Q12**	*“I like it when it is night because, it means that no one is awake and that I do not have to worry and stress about what is going to come the next day”.* *“The place where I sit to share with my family and watch TV after work”.* *“Which is a place where whenever I finish doing my homework, I lay down either to read, or just to rest”.*
**Q13**	*“That it is always better with family than alone and locked in your room”.* *“That sometimes you have to take time to relax and rest”.* *“Although we are going through difficult situations, we always have to be grateful for having a home where we can live and be with family”.*

**Table 7:** Frequency and percentage of responses with positive, negative, and neutral polarities.

Polarity	n	%
Positive	115	64.25
Neutral	38	21.22
Negative	26	14.53
Total	179	100

## Data Availability

The data are not publicly available due to ethical reasons.
